# Acknowledgement of manuscript reviewers 2014

**DOI:** 10.1186/s12944-015-0003-8

**Published:** 2015-04-30

**Authors:** Undurti Das

**Affiliations:** UND Life Sciences, United States of America, ., .

## Abstract

The editors of Lipids in Health and Disease would like to thank all our reviewers who have contributed to the journal in Volume 13 (2014).

**Tatjana Ábel**

Hungary

**Khaled Abulnaja**

Saudi Arabia

**Adeolu Adedapo**

Nigeria

**Olufunmilayo Adeleye**

Nigeria

**Roman Alberty**

Slovakia

**Suzanne Al-Bustan**

Kuwait

**Laura Allende**

United States of America

**Emma Allott**

United States of America

**María J Alonso**

Spain

**Maryam Alowayesh**

Kuwait

**Brochot Amandine**

France

**Kamal Amin**

Egypt

**Katherine Anagnostopoulou**

Greece

**Kathryn Anastos**

United States of America

**Hitoshi Ando**

Japan

**Smaragdi Antonopoulou**

Greece

**Thierry Arnould**

Belgium

**Wilbert Aronow**

United States of America

**Benoit Arsenault**

Netherlands

**Fareed Arthur**

Ghana

**Vasilios Athyros**

Greece

**Youssef Attia**

Egypt

**Alexis Baass**

Canada

**In-Hwan Baek**

Korea, South

**Camille Balarini**

Brazil

**Gabor Balogh**

Hungary

**Maciej Banach**

Poland

**Fatemeh Bandarian**

Iran

**Indranil Banerjee**

India

**Liz Andrea V. Baroncini**

Brazil

**Raul Barrera Rodriguez**

Mexico

**Nicola Basso**

Italy

**Sandip Basu**

India

**Jasmin Bavarva**

United States of America

**Jeffrey Bell**

United States of America

**Zdenka Bendova**

Czech Republic

**Matthew Benson**

Canada

**Rolf Kristian Berge**

Norway

**Alvin Berger**

United States of America

**Lars Berglund**

United States of America

**Claudio Adrián Bernal**

Argentina

**Martine S Bernstein**

Switzerland

**Stefano Bertolini**

Italy

**H Tanju Besler**

Turkey

**Sofiane Bezzine**

Tunisia

**Sanjay Bharti**

United States of America

**Seema Bhaskar**

India

**Sushant Bhatnagar**

United States of America

**Dipak Bhattacharyya**

India

**Douglas Bibus**

United States of America

**Dirk Blom**

South Africa

**Lazar Bojic**

United States of America

**Sehnaz Bolkent**

Turkey

**Sara Bonafini**

Italy

**Claudio Borghi**

Italy

**Roger Bouillon**

Belgium

**Mohammadreza Bozorgmanesh**

Iran

**Gleisson Brito**

Brazil

**Christa Buechler**

Germany

**Julia Busik**

United States of America

**Anping Cai**

China

**Philip Calder**

United Kingdom

**Diego F Calvisi**

Germany

**Catherine Calzada**

France

**Ahmet Selçuk Can**

Turkey

**Bertrand Cariou**

France

**Marin Carmen**

Spain

**Roberta Cassani**

Brazil

**Manuel Castro Cabezas**

Netherlands

**Alberico L Catapano**

Italy

**Alvaro Cerda**

Brazil

**Darko Cerne**

Slovenia

**Thais Cesar**

Brazil

**Mehervani Chaduvula**

India

**Lee-Tian Chang**

Taiwan

**Amitabha Chattopadhyay**

India

**Rima Chedid**

Lebanon

**Lanming Chen**

China

**Yanfang Chen**

United States of America

**Olga Cherepanova**

United States of America

**Adnan Chhatriwalla**

United States of America

**Eduardo Chini**

United States of America

**Agata Chmurzynska**

Poland

**Sungjin Chung**

Korea, South

**Carla Cicala**

Italy

**Mustafa Cuneyt Cicek**

Turkey

**Fernando Civeira**

Spain

**Robin Clugston**

United States of America

**Eva Corey**

United States of America

**Tamas Csont**

Hungary

**Maria Fernanda Cury-Boaventura**

Brazil

**Rongji Dai**

China

**Wenbing Dai**

China

**Dirk Dannenberger**

Germany

**Undurti Das**

United States of America

**Tommaso De Giorgis**

Italy

**Aline De Piano**

Brazil

**Tamás Decsi**

Hungary

**Hans Demmelmair**

Germany

**Zeyuan Deng**

China

**Poonamjot Deol**

United States of America

**Kine-Susann Noren Dervola**

Norway

**Paraskevi Detopoulou**

Greece

**Teresa Dias**

Portugal

**Juan Diaz-Zagoya**

Mexico

**Gordon Doig**

Australia

**Fotios Drenos**

United Kingdom

**Xiao-Jun Du**

Australia

**Qing Duan**

United States of America

**Helene Duez**

France

**Robin Dullaart**

Netherlands

**David Dyck**

Canada

**Konstantinos Economopoulos**

United States of America

**Iris Edwards**

United States of America

**Gösta Eggertsen**

Sweden

**Gudny Eiriksdottir**

Iceland

**Daisuke Ekuni**

Japan

**Robert Elkin**

United States of America

**Ibrahim Elmadbouh**

Egypt

**Matthew Elmes**

United Kingdom

**Ahmed El-Sohemy**

Canada

**Tatiana Emanuelli**

Brazil

**Munechika Enjoji**

Japan

**Sonja Entringer**

Germany

**Gokhan Eraslan**

Turkey

**Nihan Erginel-Unaltuna**

Turkey

**Abdellatif Essabbani**

France

**Debora Estadella**

Brazil

**Rhobert Wyn Evans**

United States of America

**Aldo Eynard**

Argentina

**Jane Ferguson**

United States of America

**Luiz Claudio Fernandes**

Brazil

**Theodosios Filippatos**

Greece

**Pavel Flachs**

Czech Republic

**John Flack**

United States of America

**Idumebor Florence**

Nigeria

**Bruno M Fonseca**

Portugal

**Edward Franek**

Poland

**Christian Freise**

Germany

**Michihiro Fukushima**

Japan

**Daniela Tomie Furuya**

Brazil

**Annemarie Gagnon**

Canada

**José Carlos Galduróz**

Brazil

**Alessandra Gambero**

Brazil

**Yujun Gan**

United States of America

**Xiao-Ming Gao**

Australia

**Danijela Gasevic**

Canada

**J Michael Gaziano**

United States of America

**Ingrid Gelissen**

Australia

**Christopher Gentile**

United States of America

**Marco Gentile**

Italy

**Ola Gharib**

Egypt

**M. Nabeel Ghayur**

Pakistan

**Ilias Giannenas**

Greece

**Mark Gilthorpe**

United Kingdom

**Belaineh Girma**

Ethiopia

**Julia Goedecke**

South Africa

**U Goldbourt**

Israel

**Hamidreza Goodarzynejad**

Iran

**Anilkumar Gopalakrishnapillai**

India

**Clarice Gorenstein**

Brazil

**Binita Goswami**

India

**Antonio Gotto**

United States of America

**Maria Do Carmo Gouveia Peluzio**

Brazil

**Jones Graceli**

United States of America

**Charlotte Green**

United Kingdom

**Jean-Philippe Guilloux**

France

**Grace Guo**

United States of America

**Huan Guo**

China

**Helena Gylling**

Finland

**Bodo Haas**

Germany

**Ganesh Halade**

United States of America

**Dalia Hamdy**

Egypt

**Parichehr Hanachi**

Iran

**Heather Hanwell**

Canada

**Michael Harrison**

Ireland

**Anna Haug**

Norway

**Mutlu Hayran**

Turkey

**Qi-Qiang He**

China

**Robert Hegele**

Canada

**Nazisa Hejazi**

Malaysia

**Andrea Henze**

Germany

**Emily Heying**

United States of America

**David Hill**

Canada

**Tsutomu Hirano**

Japan

**Erik Hjorth**

Sweden

**Pratik Home**

United States of America

**Byron Hoogwerf**

United States of America

**Mahmoud Hosseini**

Iran

**Ji Hu**

China

**Fenghong Huang**

China

**Anthony Hulbert**

Australia

**Jeffrey Hurst**

United States of America

**Jinah Hwang**

Korea, South

**Miki Igarashi**

United States of America

**Akihiro Inazu**

Japan

**Teruo Inoue**

Japan

**Carlos Iribarren**

United States of America

**Tetsumi Irie**

Japan

**Luigi Iuliano**

Italy

**Naoharu Iwai**

Japan

**Maria Izar**

Brazil

**Suzanne Jackowski**

United States of America

**Sonia Jancar**

Brazil

**Kamalan Jeevaratnam**

Malaysia

**Awatef Jelassi**

Tunisia

**Subrina Jesmin**

Japan

**Scott Johnstone**

United Kingdom

**Risto Kaaja**

Finland

**Takashi Kadowaki**

Japan

**Rajender Rao Kalashikam**

India

**Rajender Kalashikam**

India

**Sekhar Kambakam**

United States of America

**Atsuko Kamijo-Ikemori**

Japan

**Toshinori Kamisako**

Japan

**Tsugiyasu Kanda**

Japan

**Ken Karasawa**

Japan

**Joel Karliner**

United States of America

**Anders H Karlsson**

Denmark

**Keizo Kasono**

Japan

**Nurkay Katrancioglu**

Turkey

**Zinovia Kefalopoulou**

United Kingdom

**Theodoros Kelesidis**

United States of America

**Darshan Kelley**

United States of America

**Simon Kennedy**

United Kingdom

**Christopher Kenyon**

United Kingdom

**Mykolay Khalangot**

Ukraine

**Davood Khalili**

Iran

**Nasim Khan**

United States of America

**Nader Khandanpour**

United Kingdom

**Won Ho Kim**

Korea, South

**Elzbieta Kimak**

Poland

**Hiroyuki Kinoshita**

Japan

**Peter Kisfali**

Hungary

**Andreas Knudsen**

Denmark

**Claudia Koch-Brandt**

Germany

**Wolfgang Koenig**

Germany

**Tom Kovala**

Canada

**Czeslawa Kowal**

United States of America

**Fredric Kraemer**

United States of America

**Wolfgang Kratzer**

Germany

**Penny Kris-Etherton**

United States of America

**Malgorzata Kujawska**

Poland

**Shilpa Kulkarni**

United States of America

**Fred Kummerow**

United States of America

**Moriya Kyoji**

Japan

**Marcio Ladeira**

Brazil

**Patrick Laharrague**

France

**Jiangtao Lai**

China

**Jennifer Lambert**

Canada

**Jen Lambert**

Canada

**John Lasekan**

United States of America

**Vito Laudadio**

Italy

**Wilfried Le Goff**

France

**Séverine Ledoux**

France

**Jiyoung Lee**

United States of America

**Peng Lei**

Australia

**Marina Leitman**

Israel

**Moshe Levi**

United States of America

**Huiliang Li**

United Kingdom

**Xiao-Lin Li**

China

**Longjiang Li**

China

**Shiming Li**

United States of America

**Zeqin Lian**

China

**Evangelos Liberopoulos**

Greece

**Fabio Lima**

Brazil

**Jin-Yuarn Lin**

Taiwan

**Xiaobo Lin**

United States of America

**Lilla Lionetti**

Italy

**Guangliang Liu**

United States of America

**Monica Rosa Loizzo**

Italy

**Amedeo Lonardo**

Italy

**David Lopez**

Spain

**Lianghao Lu**

United States of America

**Qing-Hua Lu**

China

**Dieter Lütjohann**

Germany

**Yongjie Ma**

United States of America

**Makoto Makishima**

Japan

**Giovanni Malerba**

Italy

**Arul Murugan Mani**

India

**Arya Mani**

United States of America

**Prasenjit Manna**

United States of America

**Elmo Mannarino**

Italy

**Vangelis Manolopoulos**

Greece

**Enzo Manzato**

Italy

**Zehra Manzoor**

Pakistan

**Francesca Marcehgiani**

Italy

**Teodoro Marotta**

Italy

**Seth Martin**

United States of America

**Jose Martinez-Gonzalez**

Spain

**Sarabjit Mastana**

United Kingdom

**Chandra Mathela**

India

**Ahmet Mavi**

Turkey

**Robert Mcnamara**

United States of America

**Gracia Merino**

Spain

**Qi Mi**

United States of America

**Anne Marie Minihane**

United Kingdom

**Masashi Miyashita**

Japan

**Teruo Miyazawa**

Japan

**Alfredo Molinolo**

United States of America

**Wouter Moolenaar**

Netherlands

**Isabel Moreno-Indias**

Spain

**Takayuki Morisaki**

Japan

**Toshiya Morozumi**

Japan

**Lakshmi Mundkur**

India

**Harvey Murff**

United States of America

**Sohail Mushtaq**

United Kingdom

**David Mutch**

Canada

**Brent Myers**

United States of America

**Tomohiro Nabekura**

Japan

**Satoshi Nagaoka**

Japan

**Luis Cavalcante Nagato**

Brazil

**Katsuyuki Nakajima**

Japan

**Atsushi Nakajima**

Japan

**Sathish Kumar Natarajan**

United States of America

**Christopher Naugler**

Canada

**Abdul Khaliq Naveed**

Pakistan

**Mitsuru Nenoi**

Japan

**David Nerenz**

United States of America

**Flavio Nervi**

Chile

**Forrest Nielsen**

United States of America

**Toshiharu Ninomiya**

Japan

**Johnkennedy Nnodim**

Nigeria

**Giuseppe Danilo Norata**

Italy

**Begoña Ochoa**

Spain

**Da Young Oh**

United States of America

**Jude Uzoma Ohaeri**

Nigeria

**Roberta Okamoto**

Brazil

**Diogo Oliveira-Silva**

Brazil

**Claudia Maria Oller Do Nascimento**

Brazil

**Altan Onat**

Turkey

**Olayinka Oridupa**

Nigeria

**Robert Orlando**

United States of America

**Sarah Orr**

United States of America

**Fábio Orsatti**

Brazil

**Leiv Ose**

Norway

**Ali Oto**

Turkey

**William Kwame Boakye Ansah Owiredu**

Ghana

**Lila Oyama**

Brazil

**Fatih Ozcelik**

Turkey

**Ummuhani Ozel Turkcu**

Turkey

**Vladimir Palicka**

Czech Republic

**Johan Palmfeldt**

Denmark

**Wensheng Pan**

China

**Demosthenes Panagiotakos**

Greece

**Andrie Panayiotou**

Cyprus

**Ute Panzenboeck**

Austria

**Jill Parnell**

Canada

**Parul Parul**

India

**Marisa Passarelli**

Brazil

**Marcello Pastorelli**

Italy

**Jeetesh Patel**

United Kingdom

**Vandana Patravale**

India

**Gina Peloso**

United States of America

**Fabio Penna**

Italy

**Veronica Peotta**

United States of America

**Miklos Peterfy**

United States of America

**Uwe Pfeil**

Georgia

**Olivia Phung**

United States of America

**Livia Pisciotta**

Italy

**Christos Pitsavos**

Guam

**Chris Plaisier**

United States of America

**Lilian Plotkin**

United States of America

**Evangelos Polychronopoulos**

Greece

**Kalpana Poudel-Tandukar**

Japan

**Camilla Pramfalk**

Sweden

**Frederic Preitner**

Switzerland

**Indira Priyadarsini**

India

**Jose Puzo**

Spain

**M. Imran Qadir**

Pakistan

**Kemin Qi**

China

**Li Qing**

China

**Ruofeng Qui**

China

**Francisco Quintana**

United States of America

**Eder Cr Quintao**

Brazil

**Nara Quintão**

Brazil

**Tia Rains**

United States of America

**Elanchezhian Rajan**

United States of America

**Vasudevan Ramachandran**

Malaysia

**Giuliano Ramadori**

Germany

**Lakshmy Ramakrishnan**

India

**Stanley Rapoport**

United States of America

**Mathias Rask-Andersen**

Sweden

**Heather Rasmussen**

United States of America

**Lipika Ray**

United States of America

**Raylene Reimer**

Canada

**Scott Reisman**

United States of America

**Matteo Ricchi**

Italy

**Andreas Ritsch**

Austria

**Angela Albarosa Rivellese**

Italy

**Maha Rizk**

Egypt

**Manfredi Rizzo**

Italy

**Sander Robins**

United States of America

**Joao Rocha**

Brazil

**Isabel Rodriguez**

Spain

**Maricela Rodriguez-Cruz**

Mexico

**Nikki Rogers**

United States of America

**June Romeo**

United States of America

**Martin Rossmeisl**

Czech Republic

**Basil Roufogalis**

Australia

**Guy Rousseau**

Canada

**Larry Rudel**

United States of America

**Iwona Rudkowska**

Canada

**Farid Saad**

Germany

**Mohammad Saklayen**

United States of America

**Angelo Sala**

Italy

**Serafina Salvati**

Italy

**Valeria C. Sandrim**

Brazil

**Bishwa Sapkota**

Japan

**Beata Sarecka-Hujar**

Poland

**Ludger Scheja**

Germany

**Caroline Schmidt**

Sweden

**Barbara Scolnick**

United States of America

**Mark Seielstad**

United States of America

**Akira Sekikawa**

United States of America

**Ildiko Seres**

Hungary

**Itzhak Shapira**

Israel

**Sandeep Sharma**

Canada

**Shyam Sharma**

India

**Praba Sheela**

India

**Shengrong Shen**

China

**Aki Shinoki**

Japan

**Masataka Shiraki**

Japan

**Yun Hee Shon**

Korea, South

**Bob Siegerink**

Netherlands

**Aline Silva**

Brazil

**Kotryna Simonyte**

Sweden

**Stephen Smith**

United States of America

**Keith Solomon**

United States of America

**Guohua Song**

China

**Zhenyuan Song**

United States of America

**Slavica Spasic**

Serbia

**J St-Amand**

Canada

**Ken Stark**

Canada

**Aleksandra Stefanovic**

Serbia

**Pablo Stiefel**

Spain

**Welma Stonehouse**

Australia

**Marie-Pierre St-Onge**

United States of America

**Danielle Stringer**

Canada

**Christian Strupp**

Germany

**Ioannis Stylianou**

Canada

**Juan Suarez**

Spain

**M Elizabeth Sublette**

United States of America

**Yasuhiko Tabata**

Japan

**Gulten Tacoy**

Turkey

**Marija Takic**

Serbia

**Anand Tamatam**

India

**Tamotsu Tanaka**

Japan

**Sevda Tanrikulu Kucuk**

Turkey

**Maria Das Graças Tavares Do Carmo**

Brazil

**Claudio Tiribelli**

Italy

**Vishvanath Tiwari**

India

**Selma Tobudic**

Austria

**Gerald Tomkin**

Ireland

**Brian Tomlinson**

Hong Kong

**Hiroshi Tomoda**

Japan

**Santoshkumar Tota**

India

**Janet Tou**

United States of America

**Lucia Trevisi**

Italy

**Andres Trostchansky**

Uruguay

**Michael Tsai**

United States of America

**Sotirios Tsimikas**

United States of America

**Kazuhisa Tsukamoto**

Japan

**Vincenzo Tufarelli**

Italy

**Natsuo Ueda**

Japan

**Anetta Undas**

Poland

**Ondine Van De Rest**

Netherlands

**Ingrid Van Der Mei**

Australia

**Jacques Van Rooyen**

South Africa

**Michael Vansaun**

United States of America

**Hannah Vasanthi**

India

**Elisardo C Vasquez**

Brazil

**Vijayakumar Vavachan**

India

**Jelena Vekic**

Serbia

**Jorie Versmissen**

Netherlands

**Jorma Viikari**

Finland

**Marudachalam Vijayakumar**

India

**Raquel Villegas**

United States of America

**Karani Santhanakrishnan Vimaleswaran**

United Kingdom

**Shlomo Vinker**

Israel

**Nishant Visavadiya**

United States of America

**Daniele Vitorino**

Brazil

**Raj Vlanchi**

United States of America

**Clemens Von Schacky**

Germany

**Ine Waalkens-Berendsen**

Netherlands

**Michelle Walter**

United States of America

**Jianjun Wang**

United States of America

**Lirui Wang**

United States of America

**Jiancheng Wang**

China

**Rob Weijers**

Netherlands

**Hope Weiler**

Canada

**Pierre Weill**

France

**Semon Wu**

Taiwan

**Chaowu Xiao**

Canada

**Xiang Xie**

China

**Fenglian Xu**

Canada

**Ning Xu**

Sweden

**Minoru Yamakado**

Japan

**Kimiko Yamakawa-Kobayashi**

Japan

**Akira Yamasaki**

Japan

**Teruyoshi Yanagita**

Japan

**Guangdong Yang**

Canada

**Xilin Yang**

China

**Muhammad Yasin**

Pakistan

**Ali Umit Yener**

Turkey

**Ling Yi**

United States of America

**Kazushige Yokota**

Japan

**Silvio Zaina**

Mexico

**Aleksandra Zeljkovic**

Serbia

**Yutao Zhan**

China

**Qu Zhang**

United States of America

**Xinbo Zhang**

United States of America

**Xiaodong Zhang**

United States of America

**Song Zhengbo**

China

**Zhongkai Zhou**

China

**Wei-Li Zhu**

China

**Yuanjun Zhu**

China

**Giovanni Zuliani**

Italy

